# Reduced DJ-1-F1Fo ATP synthase association correlates with midbrain dopaminergic neuron vulnerability in idiopathic Parkinson’s disease

**DOI:** 10.1126/sciadv.ads3051

**Published:** 2025-06-06

**Authors:** Amina Abulimiti, Haesoo Bae, Anwaar Ali, Shanmuganathan Balakrishnan, Minou Tsujishita, Djordje Gveric, Travis S. Tierney, Elizabeth A. Jonas, Peter J. S. Smith, Steve M. Gentleman, Kambiz N. Alavian

**Affiliations:** ^1^ Department of Brain Sciences, Faculty of Medicine, Imperial College London, London, UK.; ^2^ Section of Endocrinology, Department of Internal Medicine, Yale University, New Haven, CT, USA.; ^3^ Institute for Life Sciences, School of Biological Sciences, University of Southampton, Southampton, UK.

## Abstract

Disruption in neuronal and synaptic metabolic homeostasis is a key driver of neurodegeneration in Parkinson’s disease (PD). Mitochondrial activity, biomass, and efficiency are critical to this balance. While activity and biomass are well characterized in PD pathology, mitochondrial metabolic efficiency remains insufficiently explored. Our previous studies showed that the protein product of PD-associated gene DJ-1 modulates metabolic efficiency through its interaction with the F1Fo-ATP-synthase β subunit (β-sub). Here, using proximity ligation assay (PLA), we compared mitochondrial DJ-1-β-sub association in distinct mesencephalic dopaminergic (mesDA) neuronal subpopulations and their intracellular compartments of PD and control postmortem brains. In PD brains, DJ-1-β-sub-PLA was lower than control in substantia nigra pars compacta (SNpc) somata and neurites but unchanged in ventral tegmental area (VTA) neurons. In PD and control cases, the PLA signal was reduced in distal neurites of SNpc compared to VTA neurons. These intracellular and region-specific differences suggest that impaired mitochondrial efficiency may contribute to the differential vulnerability of mesDA neurons in PD.

## Teaser

Reduced DJ-1-F1Fo ATP synthase association may impair mitochondrial efficiency and drive neuronal loss in Parkinson’s disease.

## INTRODUCTION

Parkinson’s disease (PD) is a prevalent and progressive movement disorder, characterized by bradykinesia, tremor, and rigidity, as well as several other motor and nonmotor symptoms (*1*, *2*). The motor symptoms primarily stem from the loss of mesencephalic dopaminergic (mesDA) neurons in the substantia nigra pars compacta (SNpc) (*2*, *3*). In comparison to their ventral tegmental area (VTA) counterparts, these neurons are particularly vulnerable to PD-associated neurodegeneration, especially in the early stages of the disease (*4*). The extensive axonal branching of the SNpc neurons, coupled with their distinct firing patterns, is thought to lead to an excessively high bioenergetic demand (*5*, *6*). Over time, failure of mitochondria to meet the inherently high metabolic burden of these neurons contributes to their enhanced susceptibility and is considered a key driver of neurodegeneration, especially under stress conditions (*7*–*10*). Disruption of mitochondrial energy metabolism, broadly and in the context of PD, can be due to several factors, including a decline in mitochondrial biomass, activity, or efficiency (*11*–*13*). While the former factors have been examined in postmortem studies and animal models of PD, the role of mitochondrial metabolic efficiency remains insufficiently explored.

Mitochondrial energetics relies on oxidative phosphorylation, driven by chemiosmosis, in which an active proton transfer system is coupled with adenosine 5′-triphosphate (ATP) production (*14*). This process depends on a protonmotive redox osmoenzyme system (respiratory complexes), a set of proton-linked porters, the protonmotive F1Fo adenosine triphosphatase, and the topologically closed inner membrane (*15*). The efficiency of the system relies on the formation of a proton gradient across the inner membrane and the coupling of electron transfer with phosphorylation (*14*, *15*). Uncoupling of the two processes, which reflects metabolic inefficiency, is predominantly a function of ion leak channels. The increased activity of the futile leak channels results in decreased ATP production relative to O_2_ uptake (lower P/O ratio) (*16*–*19*).

We recently described the function of an ion leak channel embedded within the F1Fo ATP synthase in neuronal metabolic efficiency and survival (*19*, *20*). Key modulators of this mechanism are neuroprotective factors Bcl-xL and DJ-1, the latter being the protein product of PD-associated gene *Park7* (*19*, *21*, *22*). Translocation of both Bcl-xL and DJ-1 to mitochondria and their interaction with the β subunit of ATP synthase lead to a decrease in the inner membrane ion leak conductance and increased P/O ratio (*19*, *21*–*23*). Conversely, reduced interaction of DJ-1 with the β subunit (β-sub) of ATP synthase results in dysfunctional cellular metabolism and loss of neuronal process extension in mesDA neurons (*21*, *22*).

Recent reports and our findings suggest that DJ-1 deficiency or disease-related mutations lead to an abnormally high state 4 respiration, where mitochondria consume oxygen, despite the absence of ATP synthesis, to compensate for proton leakage across the inner membrane, reflecting mitochondrial inefficiency (*21*, *24*) leading to degeneration of SNpc neurons (*25*, *26*). Direct addition of recombinant DJ-1 protein to patch-clamp recordings of rat brain submitochondrial vesicles reduced ion channel conductance, confirming that the interaction of DJ-1 and the F1Fo ATP synthase decreases mitochondrial proton leak (*21*). However, those studies did not explore a direct link between this mechanism of DJ-1–regulated metabolic efficiency and the vulnerability of SNpc neurons or PD pathology. In this study, we used proximity ligation assay (PLA) to examine the association of DJ-1 with the β-sub in different dopaminergic (DA) neuronal subpopulations in the postmortem brains of patients with sporadic PD. Here, we show a reduction in DJ-1-β-sub association in the SNpc of brains of patients with PD, particularly in distal neurites in the dorsal striatum, while in the VTA, this association is unchanged between control and PD brains. In control brains, VTA neurons exhibit an increasing gradient of DJ-1-β-sub association from somata to proximal to distal neurites, consistent with the known subneuronal metabolic expenditure profile, whereas SNpc neurons lack this gradient. These results suggest a fundamental difference in regulation of mitochondrial efficiency in the two neuronal subpopulations, which might play a role in neurodegeneration during the course of PD.

## RESULTS

### DJ-1-β-sub association is reduced in SNpc DA neurons of patients with PD

Loss of function mutations in DJ-1 causes an autosomal recessive, early-onset familial form of PD (*27*). Some reports suggest a role for this ubiquitously expressed protein in the sporadic cases of the disease (*28*–*31*). Building on the established metabolic phenotypes of mesDA neuronal subpopulations—particularly the high metabolic demand of SNpc neurons and their dorsal striatum projections (*5*, *9*, *10*), which may contribute to their selective vulnerability in PD—and our previous work demonstrating DJ-1 as a key regulator of neuronal metabolic efficiency, we postulated that metabolic efficiency, through this mechanism and DJ-1-β-sub association, is differentially regulated in SNpc and VTA neurons, with the association levels potentially altered in PD.

**Fig. 1.**
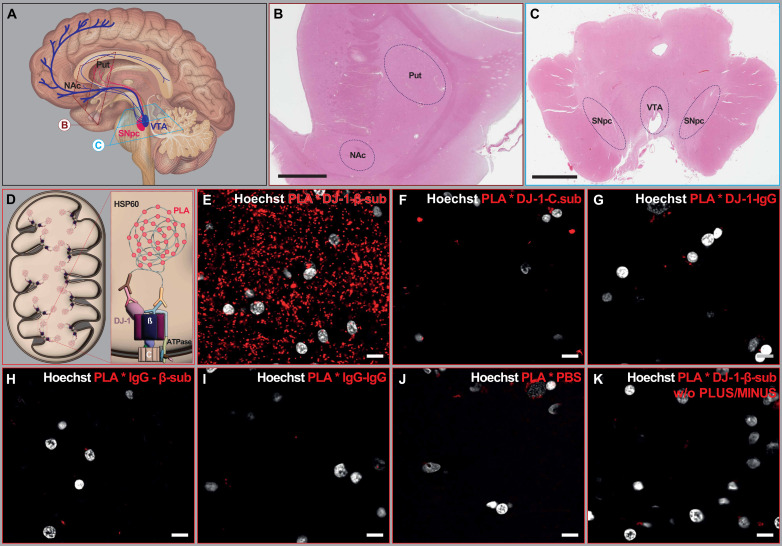
Overview of study and control experiments. (**A**) Schematic diagram illustrating the innervation map of the nigrostriatal and mesolimbic pathways, highlighting the sampled regions. (**B** and **C**) Hematoxylin and eosin (H&E) staining of the striatum (B) and midbrain (C) in human postmortem brain samples, with anatomical regions outlined. (**D**) Schematic representation of DJ-1 binding to the F1 subunit of ATP synthase in the mitochondrial matrix and the PLA assay principle. The diagram illustrates the use of primary antibodies (rabbit and mouse), PLA probes conjugated to oligonucleotides, and subsequent signal amplification for detecting DJ-1-β-sub associations. (**E** to **K**) Control experiments for the PLA assay. (E) PLA performed using primary antibodies against DJ-1 and the β-sub, showing specific signal detection. (F) PLA performed using antibodies against DJ-1 and the C-subunit of F1Fo ATP synthase. [(G) to (K)] Negative controls using DJ-1 and mouse IgG (G), Rabbit IgG and β-sub (H), Rabbit IgG-Mouse IgG (I), PBS and PLA mix (J), and PLUS/MINUS probe–excluded mix (K). Hoechst-stained nuclei are shown in gray. Scale bars, 10 μm.

**Fig. 2.**
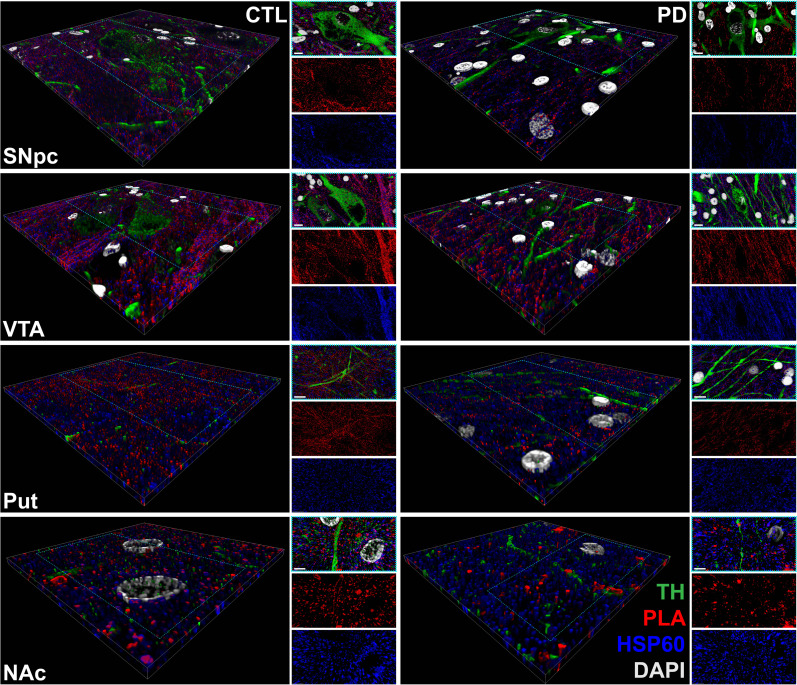
Representative 3D reconstruction of confocal images of DJ-1-β-sub PLA signal within midbrain and striatal sections. 3D reconstruction of 8-μm sections, with a magnified top view highlighting the nigrostriatal (SNpc-Put) and mesolimbic (VTA-NAc) pathways, following immunohistofluorescence. PLA puncta (red), representing DJ-1-β-sub association, were detected within HSP60-positive mitochondria (blue) of TH-positive neurons (green) and surrounding tissue in the SNpc, Put, VTA, and NAc. Hoechst-stained nuclei are shown in gray. Scale bar, 10 μm. CTL, control; DAPI, 4′,6-diamidino-2-phenylindole.

To examine the association of DJ-1 and β-sub, we used PLA, a sensitive fluorescence-based technique that detects close molecular associations by amplifying signals from antibody-bound targets in close proximity (<40 nm) (*32*). We assessed the DJ-1-β-sub PLA signal in the heat shock protein 60 (HSP60) immunoreactive mitochondria of tyrosine-hydroxylase (TH)–stained sections, within the nigrostriatal pathway, consisting of SNpc neurons in the midbrain and their projections in putamen (Put) in the dorsal striatum, as well as mesolimibic neurons, composed of VTA neurons and their projections in the nucleus accumbens (NAc) (Fig. 1, A to C). For the PLA assay, we studied a total of 20 PD [10 males and 10 females; mean age: 78.95 ± 1.61 (± denotes SEM everywhere) years] and 20 control brains (11 males and 9 females; mean age: 83.20 ± 1.87 years) (Table 1 and Fig. 1, A to C). Primary antibodies for DJ-1 and β-sub were from two different species (rabbit and mouse) (Fig. 1D). The PLA controls were performed on control brain sections by using the F1Fo ATP synthase C-subunit antibody instead of the β-sub antibody (Fig. 1F), exchanging one or both primary antibodies with isotype immunoglobulin G (IgG) from the same species (Fig. 1, G to H), replacing the antibodies with phosphate-buffered saline (PBS) (Fig. 1J), or removing PLUS and MINUS probes from the PLA mix (Fig. 1K). The representative images for experimental groups are shown in Fig. 2.

**Table 1.** Demographic and clinical characteristics of human brain cases. NPD, neuropathology diagnosis; LBDBS: Lewy body disease brainstem variant; αSN: α-synuclein; M, male; F, female.

Case	Age (years)	Sex	Onset (years)	Duration	NPD	αSN	Tau	Weight (g)
*Parkinson’s disease with Braak stage 3 pathology*								
1	69	M	56	13	LBDBS	3	2	1513
2	72	F	59	13	LBDBS	3	2	1470
3	80	M	NA	NA	LBDBS	3	2	1165
4	83	M	74	9	LBDBS	3	1	NA
5	69	F	61	8	LBDBS	3	0	1181
6	76	F	NA	NA	LBDBS	3	1	1326
7	78	M	NA	NA	LBDBS	3	1	1401
8	71	F	NA	NA	LBDBS	3	1	1089
9	79	F	NA	NA	LBDBS	3	3	1145
10	71	M	58	15	LBDBS	3	0	1243
11	80	F	70	10	LBDBS	3	2	1380
12	68	M	51	17	LBDBS	3	0	1315
13	87	M	79	21	LBDBS	3	1	1215
14	86	F	70	16	LBDBS	3	2	1366
15	86	M	70	16	LBDBS	3	0	1385
16	93	F	79	14	LBDBS	3	1	890
17	79	F	57	22	LBDBS	3	1	1245
18	80	M	58	23	LBDBS	3	2	1218
19	84	M	73	11	LBDBS	3	2	1317
20	88	F	82	7	LBDBS	3	1	1198
*Controls*								
1	80	F	–	–	0	0	2	1017
2	87	F	–	–	0	0	2	1245
3	89	M	–	–	0	0	0	1193
4	66	F	–	–	0	0	1	1211
5	87	F	–	–	0	0	2	978
6	94	F	–	–	0	0	2	1158
7	70	M	–	–	0	0	1	1352
8	82	M	–	–	0	0	2	1100
9	92	F	–	–	0	0	1	1118
10	88	M	–	–	0	0	2	1098
11	84	F	–	–	0	0	NA	1320
12	70	M	–	–	0	0	NA	1484
13	84	F	–	–	0	0	NA	1124
14	81	F	–	–	0	0	2	1106
15	68	F	–	–	0	0	P	NA
16	89	M	–	–	0	0	3	1209
17	90	M	–	–	0	0	1	1447
18	84	M	–	–	0	0	0	1154
19	91	F	–	–	0	0	3	1048
20	88	M	–	–	0	0	2	1245

**Fig. 3.**
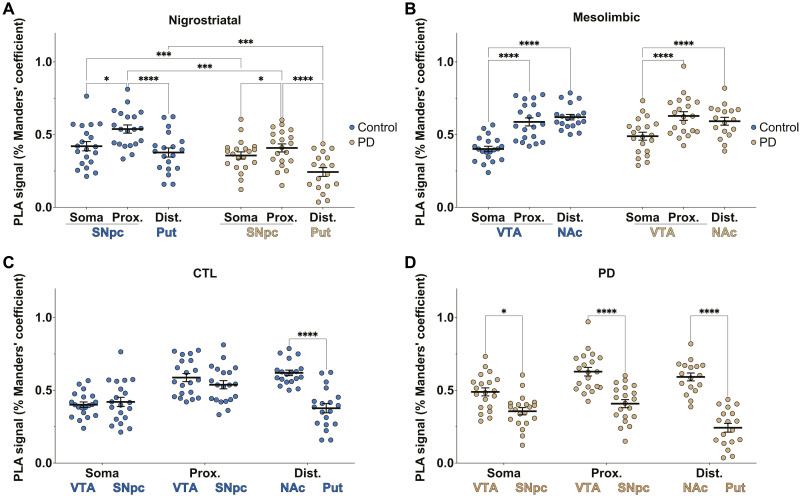
Quantification of mitochondrial DJ-1-β-sub association levels. (**A** and **B**) In situ PLA signal quantification of DJ-1-β-sub interaction within mitochondria of TH+ somata and proximal neurites in the SNpc and VTA and distal neurites in the Put and NAc, corresponding to the nigrostriatal and mesolimbic pathways, respectively, in cases of controls and patients with PD. (**C** and **D**) The data in (A) and (B) are rearranged to allow for the comparison of PLA signal within the subcellular compartments (somata, proximal neurites, and distal neurites) of mesolimbic and nigrostriatal pathways in control (C) and PD (D) cases. Each data point represents the mean of three confocal images per section (one section from each region per case). The biological replicates include 20 PD and 20 control brains (with 40 sections per group). To maintain consistency across striatal and midbrain sections, the 40 sections were processed in two independent batches of 20 sections, the maximum feasible per experimental run. Comparable trends and statistical significance were observed across both datasets. The figure presents combined data from all sections. Statistical analysis: two-way ANOVA with Tukey’s post hoc test. *P < 0.05; ***P < 0.001; ****P < 0.0001.

We found that the DJ-1-β-sub PLA signal within the mitochondria of SNpc TH-immunoreactive neurons (somata and proximal neurites) was lower in patients with PD compared to the control group. The DA axons in the Put also showed decreased DJ-1-β-sub association in brains of patients with PD compared to control cases (PD: somata *M* = 0.357 ± 0.025 R, *n* = 20; proximal neurites *M* = 0.408 ± 0.028 R, *n* = 20; Put *M* = 0.243 ± 0.030 R, *n* = 18; control: somata *M* = 0.420 ± 0.032 R, *n* = 20; proximal neurites *M* = 0.538 ± 0.028 R, *n* = 20; Put *M* = 0.377 ± 0.032 R, *n* = 19) (Fig. 3A). The DJ-1-β-sub PLA signal in VTA DA neurons showed no difference between brains of patients with PD and controls across all three subcellular regions (in VTA and NAc) (PD: somata *M* = 0.489 ± 0.027 R, *n* = 20; proximal neurites *M* = 0.628 ± 0.030 R, *n* = 20; NAc *M* = 0.592 ± 0.027 R, *n* = 17; control: somata *M* = 0.401 ± 0.019 R, *n* = 20; proximal neurites *M* = 0.588 ± 0.028 R, *n* = 20; NAc *M* = 0.620 ± 0.019 R, *n* = 19) (Fig. 3B).

The intracellular heterogeneity of mitochondria is correlated with their physiological function (*33*, *34*). Axonal and presynaptic mitochondria are specialized to meet the high energetic demand of presynaptic function. Dysfunction in these mitochondria has been suggested to contribute to neurodegenerative conditions (*35*–*39*). To determine whether the subcellular distribution of DJ-1-β-sub association levels within SNpc and VTA neurons aligns with or deviates from their expected energetic demand and expenditure, we compared the DJ-1-β-sub PLA signal levels within the SNpc and VTA DA neurons across the three subcellular regions. We observed the highest PLA signal within the mitochondria of the proximal neurites (*M* = 0.538 ± 0.028, *n* = 20), followed by distal neurites (*M* = 0.377 ± 0.032, *n* = 19) and somata (*M* = 0.420 ± 0.032, *n* = 20). A similar trend appeared in PD cases, where proximal neurites showed the highest signal (*M* = 0.408 ± 0.028, *n* = 20), followed by distal neurites (*M* = 0.243 ± 0.030, *n* = 18) and somata (*M* = 0.357 ± 0.025, *n* = 20), suggesting a correlation with subcellular metabolic demand (Fig. 3B).

In control SNpc neurons, the PLA signal does not follow the same trend observed in VTA neurons. While the PLA signal is notably higher in the proximal neurites (*M* = 0.490 ± 0.040 R, *n* = 20) compared to the somata (*M* = 0.351 ± 0.038 R, *n* = 20), it declines in the distal axons in the Put (*M* = 0.332 ± 0.047 R, *n* = 19) (Fig. 3A). In PD cases, this pattern is retained, with proximal neurites (*M* = 0.404 ± 0.046 R, *n* = 20) showing significantly higher PLA signal than the somata (*M* = 0.299 ± 0.032 R, *n* = 20), while the distal axons in the Put exhibit the lowest levels of DJ-1-β-sub association (*M* = 0.143 ± 0.034 R, *n* = 17). The trends observed in both control and PD cases indicate a mismatch in metabolic compensation along the nigrostriatal pathway, potentially contributing to the selective vulnerability of SNpc neurons in PD.

To further investigate differences in DJ-1-β-sub association between nigrostriatal (SNpc-Put) and mesolimbic (VTA-NAc) DA neurons and how these differences relate to their known vulnerability profiles, we compared the subcellular PLA signals within the same cases. In both control and PD brains, the PLA signal was higher in mitochondria (HSP60-positive) of the NAc compared to the Put, reflecting differences between the mesolimbic (VTA-NAc) and nigrostriatal (SNpc-Put) pathways. In PD brains, SNpc TH-immunoreactive somata and proximal neurites exhibited lower DJ-1-β-sub association than their VTA counterparts, whereas in control cases, no significant difference was observed between these two subcellular regions (Fig. 3, C and D—data the same as Fig. 3, A and B, rearranged to allow for this comparison). These suggest that DJ-1–mediated metabolic efficiency may contribute to the vulnerability of SNpc neurons and the proposed retrograde pattern of degeneration in PD, where synaptic and axonal dysfunction and loss precede neuronal death (*40*, *41*).

**Fig. 4.**
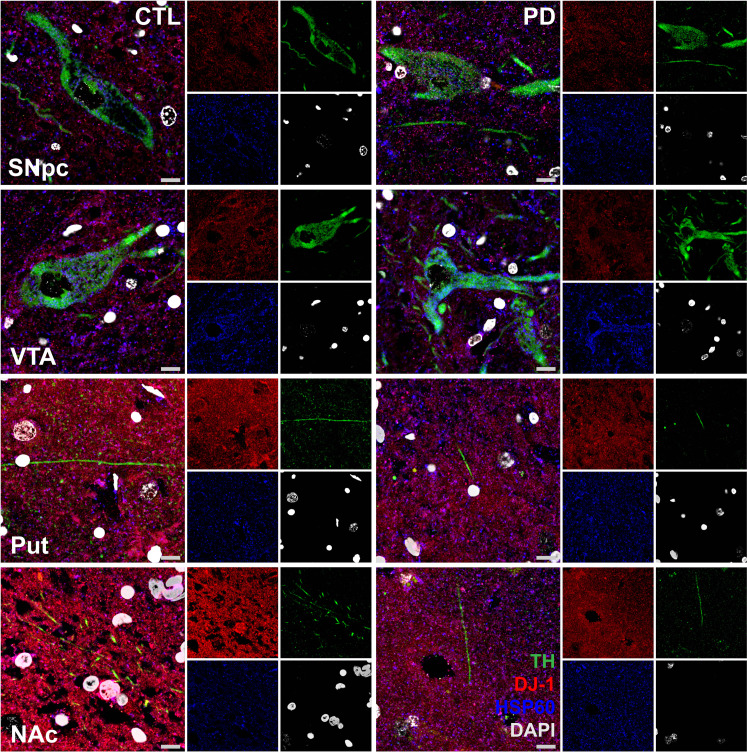
Representative confocal images of mitochondrial DJ-1 expression in control and PD brains. Immunohistofluorescence images of brain sections from controls and patients with PD, showing the SNpc, VTA, Put, and NAc regions. DJ-1 (red) is localized within HSP60-positive (blue) mitochondria of TH-immunoreactive (green) neurons and surrounding tissue. Hoechst (gray) was used to label nuclei. Scale bars, 10 μm.

**Fig. 5.**
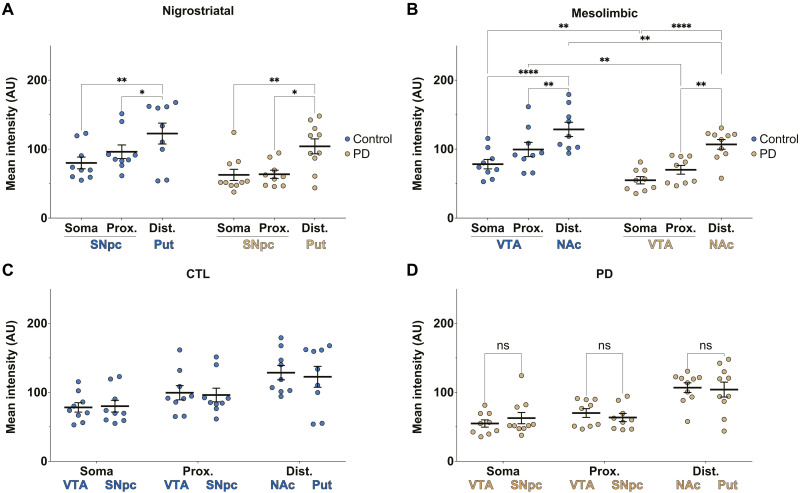
Quantification of mitochondrial DJ-1 expression in TH+ neurons and across all cell populations in control and PD brains. (**A** and **B**) Mean fluorescence intensity of DJ-1 in SNpc (A) and VTA (B) TH+ somata, proximal neurites, and distal neurites (Put and NAc). In both regions, DJ-1 levels increased from somata to distal neurites, with an overall reduction in PD cases. AU, arbitrary units. (**C** and **D**) The data in (A) and (B) are rearranged to allow for the comparison of mitochondrial DJ-1 levels within nigrostriatal (SNpc-Put) and mesolimbic (VTA-NAc) pathways in control (C) and PD (D) cases, showing no significant difference between SNpc and VTA neurons. Two-way ANOVA with Tukey’s post hoc test. *P < 0.05; **P < 0.01; ****P < 0.0001. ns, not significant.

PLA has been predominantly used to assess protein-protein interactions, using the small effective range of the assay (<40 nm), particularly when prior studies have already established the association between two proteins (*32*). In reality, the assay measures molecular proximity within a small effective range rather than direct physical interaction. Our previous studies have already demonstrated the interaction between DJ-1 and the β-sub of the F1Fo ATP synthase (*11*, *22*). Nevertheless, PLA signal differences could be due to changes in DJ-1 levels rather than to changes in its association with β-sub. Therefore, we quantified mitochondrial DJ-1 expression in the mitochondria of TH+ somata and processes of SNpc and VTA neurons using immunohistofluorescence in sections serially cut from the same regions of the same control and PD cases (Figs. 4 and 5). We also quantified the cytosolic levels of interaction between DJ-1 and 14-3-3β—an interaction we had previously shown (*22*).

In control SNpc neurons, mitochondrial DJ-1 expression levels were the lowest in somata (*M* = 79.968 ± 8.503, *n* = 9) at a medium level in proximal neurites (*M* = 96.174 ± 9.886, *n* = 9) and the highest in the distal neurites (Put; *M* = 122.584 ± 15.145, *n* = 9). A similar pattern was observed in PD cases, with DJ-1 mitochondrial expression increasing from the somata (*M* = 62.622 ± 8.128, *n* = 10) and proximal neurites (*M* = 63.522 ± 5.920, *n* = 9) to distal neurites (Put; *M* = 104.018 ± 10.836, *n* = 10) (Fig. 5A). A comparable trend was observed in VTA neurons, where mitochondrial DJ-1 levels were the lowest in somata, medium levels in proximal neurites, and the highest in the distal neurites (NAc) in both control and PD cases [control somata: *M* = 78.175 ± 6.849, *n* = 9; proximal neurites: *M* = 99.453 ± 10.332, *n* = 9; NAc: *M* = 128.558 ± 10.369, *n* = 9; PD somata: *M* = 54.833 ± 5.267, *n* = 9; proximal neurites: *M* = 70.045 ± 6.381, *n* = 9; distal neurites (NAc): *M* = 106.844 ± 6.984, *n* = 10; Fig. 5B]. In addition, the level of DJ-1-14-3-3β association was not different among the subcellular compartments of VTA and SNpc of PD or control groups (fig. S1, A and B).

The DJ-1 mitochondrial expression pattern deviates from the PLA signal trends in SNpc neurons of both PD and control groups and from that of VTA neurons in PD cases (Fig. 3, A and B). The loss of DJ-1-β-sub association in the distal neurites (Put and NAc) of SNpc neurons is not proportional to the high mitochondrial expression of DJ-1, suggesting that the PLA signal reductions are not due to decreased mitochondrial DJ-1 expression. A comparison of the mitochondrial DJ-1 levels in the TH+ neurons of nigrostriatal (SNpc-Put) and mesolimbic (VTA-NAc) DA neuron subcellular compartments also confirms this as there was no difference between the NAc and the Put or the somata and proximal neurites of SNpc versus VTA neurons in either control or PD groups (Fig. 5, C and D—data the same as Fig. 5, A and B, rearranged to allow for this comparison).

**Fig. 6.**
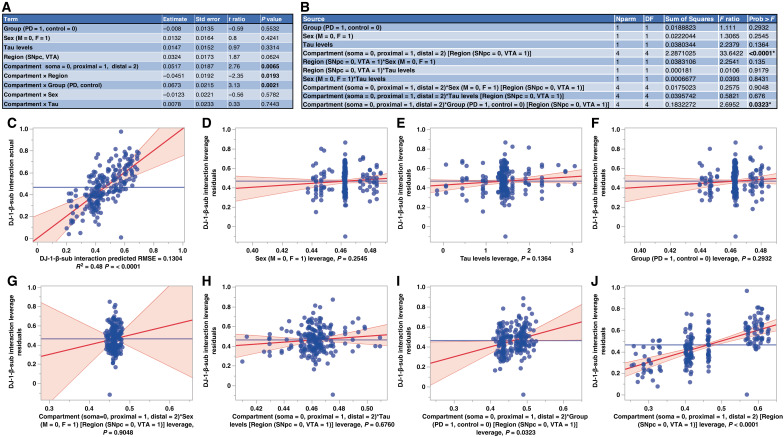
Multiple linear regression analysis of DJ-1-β-sub interaction. (**A**) The parameter estimates table summarizes regression coefficients, standard errors, *t* ratios, and *P* values, with significant effects (*P* < 0.05) observed for compartment, nested in the region (*P* = 0.0065), compartment × region (*P* = 0.0193), and compartment × group (PD versus control) (*P* = 0.0021). (**B**) The effect tests table displays the significance of main effects and interactions. Compartment, nested within the region (*P* < 0.0001), and compartment × disease (*P* = 0.0323) were significant, showing that DJ-1-β-sub association is influenced by subcellular localization, with PD affecting the association in specific subcellular compartments. (**C**) The actual versus predicted DJ-1 interaction plot illustrates the overall model fit (*R*^2^ = 0.48, RMSE = 0.1304, *P* < 0.0001), demonstrating reasonable predictive performance. (**D** to **J**) Leverage plots visualizing key predictors and interactions: (D) Sex (*P* = 0.2545), (E) tau levels (*P* = 0.1364), and (F) group (PD versus control) alone (*P* = 0.2932) do not influence DJ-1-β-sub association levels. (G) Compartment × sex × region (*P* = 0.9048) and (H) compartment × tau × region (*P* = 0.6760) show that neither sex nor tau affected DJ-1-β-sub association across compartments. (I) Compartment × group (PD versus control) (nested within the region) (*P* = 0.0323) shows a mesDA neuron subcellular compartment–specific disease effect, while (J) compartment × region (*P* < 0.0001) confirms significant variability in DJ-1-β-sub interaction across compartments within SNpc and VTA.

To explore whether sex or tau levels, in addition to disease (PD versus control), influenced DJ-1-β-sub association levels, we performed multiple linear regression analysis, incorporating the brain region (SNpc versus VTA) and subcellular compartment (soma, proximal neurites, and distal neurites, nested within the region) (Fig. 6, A and B). The model was statistically significant (*F*_22,185_ = 7.716, *P* < 0.0001), explaining 47.9% of the variance (*R*^2^ = 0.478, adjusted *R*^2^ = 0.416), with a root mean square error (RMSE) of 0.130 (Fig. 6C). The DJ-1-β-sub association was not affected by sex, tau levels, or PD versus control alone [sex (*P* = 0.2545), tau levels (*P* = 0.1364), and PD versus control (*P* = 0.2932) (Fig. 6, D to F)]. Interaction effects between compartment (nested within the region) and sex (*P* = 0.9048) or compartment (nested within the region) and tau (*P* = 0.6760) were also not significant (Fig. 6, G and H), indicating that sex and tau levels do not influence DJ-1-β-sub association within subcellular compartments across SNpc and VTA (Fig. 6, G and H). In addition, interaction effects between region and sex (*P* = 0.1350), region × tau (*P* = 0.9179), and compartment × tau (*P* = 0.6760) were not significant, indicating that DJ-1-β-sub association is not modulated by sex or tau pathology within regions or compartments.

Disease group (PD versus control) and compartment (nested within the region) interaction was significant (*P* = 0.0323), confirming that the effect of PD on DJ-1-β-sub association depends on subcellular localization of mesDA neurons (Fig. 6I). The subcellular compartment, nested within the region, had the strongest effect on the PLA signal (*P* < 0.0001) (Fig. 6J). These findings reveal that region-specific subcellular compartmentalization is the primary determinant of DJ-1-β-sub association, with the influence of PD emerging only in specific compartments rather than as a global effect.

## DISCUSSION

The present study investigates the association of DJ-1 and the β-sub of F1Fo ATP synthase in the subpopulations of mesDA neurons, which exhibit varying degrees of susceptibility to degeneration in PD. This specific association directly regulates the ion leak channel within the F1Fo ATP synthase, maintaining the separation of charge across the inner membrane. Regulation of the proton and ion gradients is essential for ATP synthesis and for the exchange of substrates required for tricarboxylic acid cycle activity, and these gradients regulate functions such as mitophagy and anaplerosis (*42*–*44*). Maintenance of electrochemical gradients also influences neuronal and synaptic growth, survival, plasticity, and regeneration. Mitochondrial dysfunction has been previously linked with neurodegeneration in PD. We reported that the DJ-1-β-sub interaction closes an ATP synthase–associated leak channel within the inner membrane and influences cellular metabolic efficiency and neuronal function (*21*, *22*).

Here, we demonstrate that DJ-1-β-sub association is selectively reduced in the subcellular compartments of SNpc of brains of patients with PD, particularly in distal neurites in the Put, while remaining unchanged in the VTA. In control brains, VTA neurons exhibit a gradient of DJ-1-β-sub association that increases from somata to proximal and distal neurites, whereas SNpc neurons lack this gradient. These patterns of DJ-1-β-sub interaction within mesDA neurons and their processes in Braak stage 3 patients, where the disease is believed to have infiltrated the forebrain and SNpc but not the VTA, suggest dysfunction in this key metabolic efficiency mechanism as an early marker and a determinant of neuronal vulnerability in PD.

The divergence between the patterns of mitochondrial DJ-1-β-sub association and DJ-1 expression underscores the potential significance of this mechanism. The finding that the decrease in distal neurites and the PD-related reduction were specific to the PLA signal and not correlated with mitochondrial DJ-1 levels suggests that DJ-1, in modulating metabolic efficiency, may play a prominent role in mitigating the differential vulnerability of mesDA neurons and PD pathogenesis in addition to its known functions in the oxidative stress response and mitochondrial quality control.

## MATERIALS AND METHODS

### Brain sample preparation and processing

All tissue samples analyzed in this study were obtained, along with associated clinical and neuropathological data, from the Parkinson’s UK Brain Bank, which is funded by Parkinson’s UK, a registered charity in England and Wales (258197) and Scotland (SC037554). All donors provided informed consent in accordance with the standard protocols of the Parkinson’s UK Brain Bank. A total of 40 postmortem brain samples was analyzed, comprising 20 individuals diagnosed with PD and 20 age-matched control subjects without neurodegenerative disease. The PD cohort consisted of 10 males and 10 females, with a mean age of 78.6 ± 6.8 years, while the control cohort included 9 males and 11 females, with a mean age of 83.5 ± 5.7 years. Brain samples were collected and processed following standardized protocols of Parkinson’s UK Brain Bank, ensuring consistency with prior studies [including our work (*11*)] that have used these samples.

Cases were selected on the basis of detailed neuropathological assessments, ensuring that all PD cases exhibited Braak stage 3 Lewy body pathology and that no individuals had significant Alzheimer’s disease–related tau pathology beyond Braak stage 3, ensuring that tau burden is not a major confounding factor in our analysis. Tau pathology scores were obtained from the Parkinson’s UK Brain Bank using their standardized neuropathological assessment, following Braak staging and NIA-AA criteria and using immunohistochemistry with an antibody against phosphorylated tau (AT8 clone; MN1020; Thermo Fisher Scientific; 1:600), as described previously (*45*). Postmortem brains were hemisected, with one hemisphere fixed in 10% neutral buffered formalin for at least 3 weeks before being paraffin embedded. All brain tissue was formalin fixed and paraffin embedded (FFPE), with 8-μm-thick midbrain and striatal sections containing SNpc, VTA, Put, and NAc regions (Fig. 1, B and C) prepared for subsequent immunohistofluorescence and PLA. To maintain consistency across striatal and midbrain sections, the 40 sections were processed in two independent batches of 20 sections, the maximum feasible per experimental run. The biological replicates include 20 PD and 20 control brains (40 sections per group). For quantification of mitochondrial DJ-1 levels, one of the sets (10 PD and 10 control sections) was specifically analyzed under identical experimental conditions, ensuring comparability and reproducibility across the study.

### Immunohistofluorescence and PLA

Immunohistochemistry was performed on 8-μm FFPE sections using established protocols with modifications (*11*, *46*). The FFPE sections were preheated for 30 min at 62°C, then deparaffinized by three successive 10-min baths in xylene, and rehydrated in decreasing grades of alcohol. Heat-induced epitope retrieval was carried out using tris-EDTA (pH 8.0) buffer, followed by blocking with 10% normal serum and 0.3% Triton X-100 in PBS for 1 hour at room temperature. The sections were then incubated in a solution containing primary antibodies for 72 hours at 4°C on a shaker, followed by overnight incubation in a solution containing a fluorophore-conjugated secondary antibody specific to the primary antibody species. PLA was then performed on the immunohistochemistry-labeled sections. We conducted in situ PLA experiments using the Duolink Kit (Sigma-Aldrich) according to the manufacturer’s protocol. Briefly, immunofluorescence-labeled tissue samples were incubated overnight at 4°C with primary antibodies (Rabbit anti-DJ1, 1:100, Allele; and Mouse anti-β-sub, 1:100, Thermo Fisher Scientific). Samples were then incubated with secondary antibodies conjugated with oligonucleotides (PLA and MINUS probes). Samples were then incubated with ligation solution to connect the two PLA probes if the probes were within proximity (<40 nm apart). The amplification solution containing polymerase then initiated rolling-circle amplification to create a PLA signal detectable by fluorescence. Last, samples were stained with Hoechst for 30 min at room temperature. Table S1 contains detailed information about the antibodies and reagents used. Key PLA troubleshooting and optimization steps are included in table S2.

### Imaging and quantification

The immunofluorescence signal was visualized using the Leica STELLARIS 8 Inverted Confocal Microscope with a 63× oil immersion objective. Images were captured with regions of interest containing one to three midbrain DA neurons (three images per case) or where the greatest number of striatal fibers could be found and processed using the Fiji/ImageJ colocalization analysis tool, JACoP, or our automated approach for quantification of puncta or mean intensity of the signal. The analysis was automated by incorporating JACoP into a custom-written ImageJ macro (available upon request), which measured the overlapping ratio of PLA in HSP60 within TH-positive somata and processes. Manders’ coefficient was used to determine the proportion of the PLA puncta that coincided with the HSP60 channel signal over its total intensity (*47*). After background subtraction using a rolling ball algorithm (radius, 50 pixels), we evaluated the pixel intensity distribution within the TH-positive areas (soma and processes) to define the threshold for the PLA signal. The intensity threshold for the PLA channel was set to 120 to 255 (arbitrary units), with 120 representing the lower limit of detection. We chose these values following an approach similar to that described by Manders *et al.* (*48*) and Costes *et al.* (*49*), where we initially assessed the cytofluorogram to identify the separation between background noise and true signal. We then performed iterative testing, comparing thresholded images with the raw data, to ensure that the puncta with intensities above 120 corresponded to the expected structural features and that background fluorescence was minimized. Negative control images (Fig. 1, F to K) were used to confirm that signals detected above this threshold were specific and eliminated false positive detections. Representative three-dimensional (3D) images were reconstructed using Leica LASX software.

We used mean intensity for comparison of DJ-1 levels in all subcellular compartments and Manders’ coefficient, which integrates both signal intensity and colocalization and is particularly well suited for measuring the high-intensity, well-defined, and aggregated mitochondrial PLA signal. For the diffuse cytosolic signal, which does not lend itself to an intensity-overlap metric, we counted discrete PLA puncta per cytosolic area.

### Statistical analyses

The data were analyzed using GraphPad Prism and are presented as the means ± SEM. The biological replicates and statistical significance are specified in the text, figures, and figure legends. Statistical analyses were performed with two-way analysis of variance (ANOVA) and Tukey’s post hoc test using GraphPad Prism. We performed multiple linear regression analysis in JMP Pro (version 17.2.0) using the standard least squares method to assess the effects of sex, tau levels, disease group (PD versus control), brain region, and subcellular compartment on DJ-1-β-sub association. Subcellular compartment (somata, proximal neurites, and distal neurites) was nested within the region (SNpc and VTA), and interaction terms (region × sex, region × tau, compartment × sex, compartment × tau, and compartment × disease group) were included. To optimize model fit, we applied the Akaike information criterion, followed by diagnostics for multicollinearity (variance inflation factors) and residual independence (Durbin-Watson test). Model assumptions were assessed using residual plots, Q-Q plots, and leverage diagnostics, while adjusted *R*^2^, RMSE, and *F*-statistics (*P* < 0.05) were used to evaluate performance. For visualization, leverage plots were used to examine predictor effect and actual versus predicted plots were generated to assess model fit (*50*).

### Ethical approval

Parkinson’s UK Brain Bank brain donations and research protocols have been approved by the relevant Research Ethics Committees (ethics reference number: 23/WA/0273).
