# Supplementary material for: Reduced DJ-1-F1Fo ATP synthase association correlates with midbrain dopaminergic neuron vulnerability in idiopathic Parkinson’s disease
Source: Sci Adv. 2025 Jun 6;11(23):eads3051. doi: 10.1126/sciadv.ads3051 (PMC12143374; doi:10.1126/sciadv.ads3051)
Supplement: Supplementary file 1 — Fig. S1 Tables S1 and S2 [file sciadv.ads3051_sm.pdf]

Science Advances
AAAS

Supplementary Materials for

Reduced DJ-1-F1Fo ATP synthase association correlates with midbrain dopaminergic neuron vulnerability in idiopathic Parkinson's disease

Amina Abulimiti *et al.*

Corresponding author: Kambiz N. Alavian, k.alavian@imperial.ac.uk

Sci. Adv. **11**, eads3051 (2025)
DOI: 10.1126/sciadv.ads3051

This PDF file includes:

Fig. S1
Tables S1 and S2

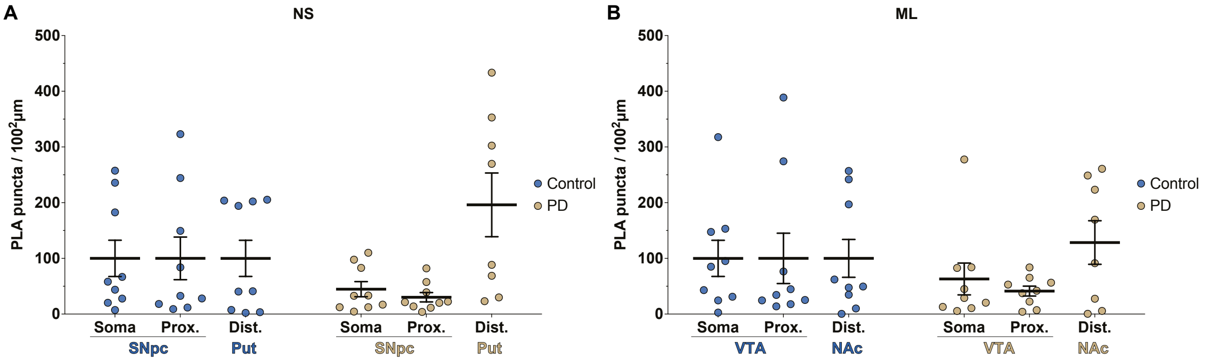

Figure S1. Quantification of DJ-1–14-3-3β PLA signal across SNpc and VTA neurons in control and PD cases. (A, B) In situ PLA signal quantification, representing DJ-1–14-3-3β association in TH+ somata, proximal neurites, and distal neurites within the nigrostriatal (NS: SNpc-Put) and mesolimbic (ML: VTA-NAc) pathways in control and PD patient cases. (A) SNpc neurons exhibit no significant differences in DJ-1–14-3-3β association between control and PD cases across subcellular compartments. (B) Similarly, in VTA neurons, DJ-1–14-3-3β association remains comparable between groups. Each data point represents the mean number from three confocal images per section. PLA puncta per 100 µm², with measurements taken from TH+ neurons in midbrain and striatal sections (one section from each region per case). Data are presented as mean ± SEM.

	Source	Identifier	Concentration
Antibodies and reagents			
Chicken anti-TH polyclonal antibody	GeneTex	GTX85470	1:100
Rabbit anti-HSP60 polyclonal antibody	GeneTex	GTX110089	1:100
Rabbit anti-DJ-1 polyclonal antibody	Allele	ABP-PAB-24369	1:100
Mouse anti-ATP synthase β monoclonal antibody	Thermo Fisher	MA1-930	1:100
Mouse anti-DJ-1 monoclonal antibody	Santa Cruz Biotechnology	sc-55572	1:100
Rabbit anti-14-3-3 β polyclonal antibody	Santa Cruz Biotechnology	sc-628	1:100
Rabbit anti-IgG polyclonal antibody	Biolegend	910801	10 μg
Mouse anti-IgG monoclonal antibody	Biolegend	401402	10 μg
FITC Goat anti-chicken IgY secondary antibody	Biolegend	410802	1:500
Donkey anti-rabbit IgG-CFL 647	Santa Cruz Biotechnology	sc-362291	1:500
Duolink In Situ PLA Probe Anti-Mouse Plus	Sigma-Aldrich	DUO92001	-
Duolink In Situ PLA Probe Anti-Rabbit Minus	Sigma-Aldrich	DUO92005	-
Duolink In Situ Detection Reagents Orange	Sigma-Aldrich	DUO92007	-
Duolink In Situ Wash Buffer A	Sigma-Aldrich	DUO82046	-
Duolink In Situ Wash Buffer B	Sigma-Aldrich	DUO82048	-
Duolink In Situ Mounting Medium with DAPI	Sigma-Aldrich	DUO82040	-
Hoechst	Sigma-Aldrich	B2261	5 μg/mL

Table S1. List of antibodies and reagents and their relevant information.

	Primary Antibody	Secondary Antibody	Baking	Heat induction	Antigen Retrieval solution	Blocking
	TH (Cell Signaling Technology, 58844S)	FITC Goat anti-chicken IgY secondary antibody (Biolegend, 410802)	Oven	Steam cooker	Sodium Citrate pH6.0	10% Goat Serum + PBST(0.2%TritonX-100)
	TH (Atlas, AMAB91112)	Donkey anti-rabbit IgG-CFL 647 (Santa Cruz Biotechnology, sc-362291		Microwave	EDTA pH10.0	10% Donkey Serum + PBST (0.3%TritonX-100)
	TH (Raybiotech, 114-10061)	Goat anti-Rabbit IgG, Alexa Fluor 647 (Thermo Fisher Scientific, A-21244)			EDTA pH8.0	10% Goat Serum + PBS
	TH AlexaFluor 488 (BioLegend, 818005)					10% Fetal bovine serum + PBST(0.3%TritonX-100)
	TH (GeneTex, GTX85470)					10% Goat Serum + PBST(0.3%TritonX-100)
	HSP60 (GeneTex, GTX110089)					
	Park 7/DJ-1 (GeneTex, GTX132552)					
	DJ-1 (Allele, ABP-PAB-24369)					
	DJ-1(D29E5)(Cell Signaling Technology, 5933s)					
	DJ-1 (Invitrogen, PA5-80881)					
	DJ-1 (Santa Cruz Biotechnolgy, sc32874)					
	ATP Synthase Beta (Invitrogen, MA1-930)					
Dilution Factors	1:200	1:400	-		-	-
	1:100	1:500				
	1:50	1:1000				
Antibody Diluent	10% Goat Serum+1xPBST(0.3%TritonX-100)	10% Goat Serum+1xPBST(0.3%TritonX-100)	-		-	-
Incubation Time			30mins			
	12-16 hrs	1hr	45mins		30mins	2hrs
	24 hrs	24hrs	1hr		45mins	1hr
	72hrs	48hrs	2hrs		1hr	45mins
			50°C			
Temperature	4 °C	RT	60°C	95–100°C.	95–100°C.	RT

Table S2. Summary of key PLA optimisation and troubleshooting steps. Light blue indicates the final reagent or optimised condition.
